# First report of chronic implant-related septic arthritis and osteomyelitis due to *Kytococcus schroeteri* and a review of human *K. schroeteri* infections

**DOI:** 10.1007/s15010-012-0250-9

**Published:** 2012-03-06

**Authors:** J. F. W. Chan, S. S. Y. Wong, S. S. M. Leung, R. Y. Y. Fan, A. H. Y. Ngan, K. K. W. To, S. K. P. Lau, K.-Y. Yuen, P. C. Y. Woo

**Affiliations:** 1 Department of Microbiology, Queen Mary Hospital, Pokfulam, Hong Kong SAR China; 2 State Key Laboratory of Emerging Infectious Diseases, The University of Hong Kong, Pokfulam, Hong Kong SAR China; 3 Research Centre of Infection and Immunology, The University of Hong Kong, Pokfulam, Hong Kong SAR China; 4 Carol Yu Centre for Infection, The University of Hong Kong, 102 Pokfulam Road, Pokfulam, Hong Kong SAR China

**Keywords:** *Kytococcus schroeteri*, Arthritis, Osteomyelitis, Implant, Infection

## Abstract

We report the first case of *Kytococcus schroeteri* implant-related septic arthritis and osteomyelitis, identified by phenotypic tests and 16S rRNA sequencing, which responded to implant removal and doxycycline. 16S rRNA sequencing was useful for the accurate and rapid identification of the organism as it exhibited three different colonial morphologies in vitro.

## Introduction

The genus *Kytococcus*, literally meaning “a coccus from skin”, was separated from *Micrococcus* spp*.* in 1995 based on phylogenetic and chemotaxonomic properties [1]. Kytococci are Gram-positive, pigmented, non-encapsulated, non-motile, aerobic, catalase-positive cocci in pairs or tetrads. The genus now consists of three species: *K. sedentarius*, *K. schroeteri*, and *K. aerolatus*. Sporadic cases of prosthetic valve endocarditis and pneumonia in immunocompromised patients due to *K. schroeteri* have been described, but to the best of our knowledge, we report here the first case of chronic implant-related septic arthritis with contiguous osteomyelitis caused by a strain of *K. schroeteri* which exhibited three different colonial morphologies in vitro.

## Case report

A 45-year-old Chinese man who suffered from left shoulder dislocation requiring tendon reconstruction with a silicone graft 15 years previously presented with recurrent flare-ups of chronic left shoulder infection manifesting as increasing pain and discharge. He first experienced left shoulder pain and discharge 6 years earlier, at which time a firm subcutaneous mass at the left acromioclavicular joint and a discharging sinus at the coracoid process of the left scapula were detected on physical examination. The roentgenogram showed an old fracture with callus formation at the distal left clavicle. Ultrasonography of the left shoulder showed an inflammatory mass measuring 46 × 9 × 20 mm in the subcutaneous tissues overlying the acromioclavicular joint. This mass communicated with a second mass overlying the coracoid process, the lateral aspect of the pectoralis muscle, and extended to the glenohumeral joint. A sinogram detected a sinus tract, approximately 7 cm in length, which extended superomedially, with its cephalic end located just below the coracoid process of the left scapula. Culture of the debrided wound tissue yielded methicillin-sensitive *Staphylococcus aureus*, and the patient was treated with a 2-week course of cloxacillin. However, his symptoms recurred 1 year later. Computerized tomography scan of the left shoulder showed diffuse bony sclerosis and pus in the sinus tract extending from the skin surface to the old fracture site at the distal left clavicle and left acromioclavicular joint. Repeated debridement was performed, and culture of the debrided tissue yielded “*Micrococcus* spp*.*” Despite initial response, the patient’s condition worsened again after another 2 years. Magnetic resonance imaging showed both an enhancing lesion in the left distal clavicle extending into the overlying subcutaneous fat and a non-enhancing mass, suspected to be surgical material, within the lesion. In view of the recurrent symptoms despite repeated wound debridement and cloxacillin, a more extensive operation consisting of ostectomy, joint lavage, wound debridement, and removal of the implanted silicone graft, was performed.

**Fig. 1:**
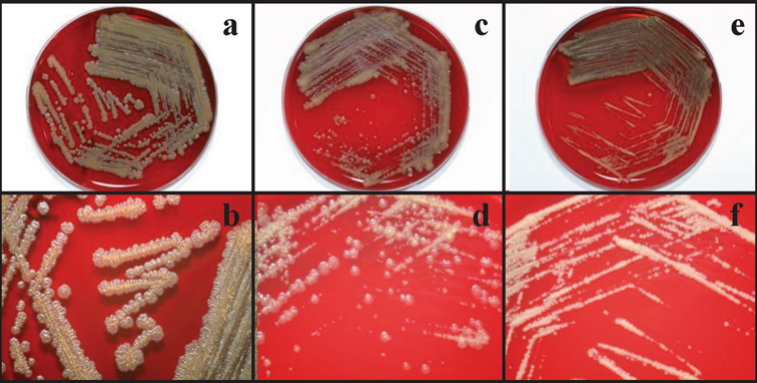
Appearances of *Kytococcus schroeteri* morphotypes 1, 2, and 3 in 5% sheep blood agar after 72 h of incubation in 5% CO_2_ at 37°C. **a**, **b** Morphotype 1, **c**, **d** morphotype 2, **e**, **f** morphotype 3

Aerobic culture of the debrided bone yielded tiny non-hemolytic colonies of Gram-positive cocci in tetrads and clusters on 5% sheep blood agar after 24 h of incubation in 5% CO_2_ at 37°C. After 48–72 h of incubation, colonies with three different types of morphologies became apparent on macroscopic observation (Fig. 1). Morphotype 1 was muddy yellow, dry, rough, and volcano-like, had irregular edges, and measured about 2.5 mm in diameter. Morphotype 2 had a lighter yellow color and smooth edges, was pleomorphic, and measured about 0.5–1.5 mm in diameter. Morphotype 3 had a chalky yellow color, was tiny, and measured about 0.5–1 mm in diameter. All three morphotypes tested positive for catalase, arginine dihydrolase, alkaline phosphatase, and pyrazinamidase, and negative for oxidase, lecithinase, β-galactosidase, and urease. They hydrolyzed gelatin and Tween 80, but not esculin. The API Rapid ID 32 Strep (bioMérieux, Marcy l’Etoile, France) and the BD Phoenix automated microbiology (Becton–Dickinson Diagnostics, Sparks, MD) systems identified all three morphotypes to be “*Micrococcus* spp*.*” with 99% confidence levels. Scanning electron microscopy, performed as described in our previous publications [2, 3], showed spherical cells measuring around 1.0 μm in tetrads or clusters.

**Fig. 2:**
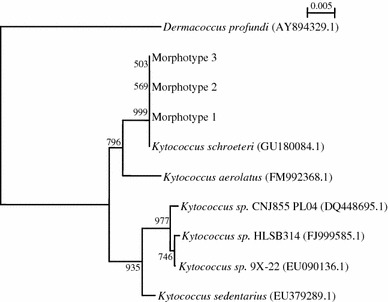
Phylogenetic tree showing the relationships of the three morphotypes to closely related species. The tree was inferred from 16S rRNA gene sequence data by the neighbor-joining method and rooted using the 16S rRNA gene sequence of *Dermacoccus profundi* (AY894329.1). Bootstrap values were calculated from 1,000 trees. The *scale bar* indicates the estimated number of substitutions per 200 bases. Names and accession numbers are given as cited in the GenBank database

In view of their phenotypic differences from micrococci, namely, resistance to penicillin and oxacillin, and positive arginine dihydrolase activity, 16S rRNA gene sequencing was performed, as described in our previous publications for other Gram-positive cocci [4–6], using LPW1282 5′-GCGTGCTTAACACATGCAAG-3′ and LPW58 5′-AGGCCCGGGAACGTATTCAC-3′ (Sigma-Proligo, Singapore) as the PCR and sequencing primers. The sequences of the PCR products were compared with sequences of closely related species in the GenBank by multiple sequence alignment using ClustalX 1.83 [7], and phylogenetic relationships were determined using the neighbor-joining method. Sequencing of the 16S rRNA gene of the morphotypes showed that the 16S rRNA gene sequences of the three morphotypes were identical. There was no base difference between the 16S rRNA gene sequence of the three morphotypes and that of *K. schroeteri* (GenBank Accession No. GU180084.1), 12 (1.3%) base differences between the 16S rRNA gene sequence of the morphotypes and that of *K. aerolatus* (GenBank Accession No. FM992368.1), and 15 (1.6%) base differences between the 16S rRNA gene sequence of the morphotypes and that of *K. sedentarius* (GenBank Accession No. EU379289.1), confirming that all three morphotypes were *K. schroeteri* (Fig. 2).

Antimicrobial susceptibility tests were performed by the disk diffusion method (Bio-Rad, Hercules, CA) on Mueller–Hinton agar. The results were expressed as susceptible, intermediate, or resistant according to the criteria of the Clinical and Laboratory Standards Institute (CLSI) for staphylococci [8]. All three morphotypes were resistant to penicillin, oxacillin, cefuroxime, clindamycin, and fusidic acid and were susceptible to amoxicillin/clavulanate, minocycline, rifampicin, and vancomycin.

Postoperatively, the patient was treated with a 6-week course of oral doxycycline 100 mg every 12 h. He had good clinical response, and the symptoms and raised inflammatory markers resolved after 1 week. He had remained well at the follow-up 3 years after the operation.

## Discussion

Traditionally, the different colonial appearances among the three *Kytococcus* spp. is considered to be a direct and helpful property by which to differentiate them. Whilst *K. sedentarius* is classically described as having a “deep buttercup yellow” or “cream-white” color [1], *K. schroeteri* is known for its “muddy yellow” pigmentation [9]. *K. aerolatus*, which has yet to be implicated in human infection, has a beige pigmentation [10]. Our case describes a novel and important laboratory observation of *K. schroeteri*: the existence of different colonial morphologies within the same organism*.* As shown in Fig. 1, colonies with three different macroscopic morphologies were isolated from our patient. In this situation, the macroscopic appearance of the colonies was not reliable tool to differentiate the organisms from other pigmented *Kytococcus* spp. Although various physiological properties and fatty acid compositions may be useful to separate the three species of *Kytococcus*, the results are often inconclusive and the analyses time-consuming. In contrast, the application of 16S rRNA gene sequencing allows rapid and accurate identification of the organisms. Using this technique, we identified all three morphotypes from our patient as *K. schroeteri*. Taking into account their similar scanning electron microscopic appearances, biochemical properties, and identical 16S rRNA gene sequences, we concluded that all three morphotypes belonged to the same strain of *K. schroeteri* and that this strain possessed different colonial morphologies.

In addition to generating new observations on the organism’s microbiological characteristics, our case also carries significant clinical impact in being the first case of *K. schroeteri* chronic implant-related septic arthritis with contiguous osteomyelitis. After its first isolation from the blood of a patient with prosthetic valve endocarditis in 2002 [9, 11], the clinical significance of *K. schroeteri* has been increasingly recognized in the past decade. Including our case, a total of 14 cases of *K. schroeteri*-related infections have been reported in the literature (Table 1), namely, six (43%) cases of prosthetic valve endocarditis [9, 11–16], five (36%) cases of pneumonia [17–20], one (7%) case of ventriculoperitoneal shunt infection [21], one case (7%) of folliculitis [19], one case (7%) of infective spondylodiscitis [22], and our case of chronic implant-related septic arthritis with contiguous osteomyelitis. The most common site of isolation of the organism was the blood (9/14; 64%), followed by respiratory tract secretions (5/14; 36%), bone (2/14; 14%), prosthetic heart valve (1/14; 7%), and cerebrospinal fluid (1/14; 7%). Among the patients who did not have an infection of a prosthesis, the majority (5/6; 83%) had an immunocompromised state (use of prednisolone or acute myeloid leukemia). The other patient with lumbar spondylodiscitis developed the infection after an operation which compromised the local immunity [22]. Thus, similar to coagulase-negative staphylococci and micrococci, *K. schroeteri* is regarded as an opportunistic pathogen which is capable of causing prosthesis-related infections, skin infection, osteomyelitis, and fatal pneumonia in immunocompromised hosts. The prognosis of patients with *K. schroeteri*-related infections mainly depend on the clinical presentation and immune status of the patient. All five patients with pneumonia were immunocompromised, and the majority of them (4/5; 80%) died within 1 month despite supportive treatment. The nine patients with other forms of clinical manifestations (infection of prostheses or lumbar spondylodiscitis) recovered with antibiotics and surgical removal of prostheses. Unlike coagulase-negative staphylococci and micrococci, however, the natural habitat of *K. schroeteri* has not been confirmed to be the human skin and mucous membranes. Szczerba and Krzeminski [23] isolated *K. sedentarius*, but not *K. schroeteri*, from human skin and mucous membranes. Le Brun [12] and colleagues were also unable to recover *K. schroeteri* from the skin and mucous membranes of their patient. However, at the time of Szczerba and Krzeminski’s study, *K. schroeteri* was probably unrecognized. Thus, further sampling should be performed to assess whether *K. schroeteri*, like *K. sedentarius*, is part of the skin and mucous membranes flora.

**Table 1:** Characteristics of patients with infections due to *Kytococcus schroeteri*

Reference	Sex/age and predisposing factor(s)	Clinical syndrome (presentation)	Site of isolation	Treatment	Outcome
Becker et al*.* [9, 11]	F/34 Aortic dissection with implantation of aortic arch conduit and reimplantation of the supraaortic arteries 10 weeks previously	Prosthetic valve endocarditis (fever, TOE showed paravalvular abscess and vegetation, septic emoblic stroke with right-sided hemiparesis)	Blood	Antibiotics (IV vancomycin, gentamicin and rifampicin for 3 weeks) followed by aortic arch prosthesis replacement	Discharged after admission
Le Brun et al. [12]	M/73 years AVR 3 years previously	Prosthetic valve endocarditis (fever, exertional dyspnea, TOE showed small vegetations on the aortic bioprosthesis and perivalvular abscess)	Blood, vegetation, perivalvular abscess, and prosthetic valve	AVR and antibiotics (IV vancomycin and then teicoplanin for 6 weeks, gentamicin and rifampicin for the initial 3 weeks)	Discharged after clinical remission
Mohammedi et al. [17]	F/71 years Asthma on prednisone 20 mg daily Hypertension	Bacteremic community-acquired pneumonia (fever, respiratory distress with right lower lobar pneumonia)	Blood and BAL fluid	Supportive treatment (bronchodilators, steroid, magnesium sulfate and antibiotics including IV ceftriaxone and ofloxacin)	Died 4 days after admission to ICU due to refractory septic shock and multiorgan failure
Mnif et al. [13]	F/49 years MVR 10 years previously	Prosthetic valve endocarditis (fever, TOE showed prosthesis disinsertion and vegetations on the mitral valve prosthesis)	Blood	MVR and antibiotics (IV pristinamycin, vancomycin and gentamicin for 6 weeks then oral rifampicin and pristinamycin for 3 weeks)	Discharged after clinical remission (6 weeks after admission)
Renvoise et al. [14]	M/70 AVR	Prosthetic valve endocarditis (fever, TOE showed vegetation on the prosthetic aortic valve and intertrigonal abscess, septic embolic stroke and bilateral renal emboli)	Blood	Antibiotics (IV amoxicillin for 6 weeks and gentamicin for the initial 2 weeks) followed by AVR 3 months afterwards	Discharged after clinical remission
Aepinus et al. [15]	F/38 AVR twice Ventricular septal defect with surgical closure Diabetes mellitus	Prosthetic valve endocarditis (fever, TOE showed vegetation on the prosthetic aortic valve)	Blood	Antibiotics (IV vancomycin and rifampicin for 6 weeks, gentamicin for the initial 2 weeks; followed by oral levofloxacin and rifampicin for 2 months)	Discharged after clinical remission
Jourdain et al. [21]	M/13 months Cyanotic congenital heart disease Hydrocephalus with VP shunt 5 months previously	VP shunt infection (fever, acute otitis media, abnormal CSF findings)	CSF	Removal of shunt and antibiotics (IV vancomycin and rifampicin for 27 days)	Discharged after clinical remission
Poyet et al. [16]	M/72 AVR for aortic regurgitation Triple bypass for ischemic heart disease	Prosthetic valve endocarditis (fever, septic embolic stroke, TTE showed vegetation on the prosthetic aortic valve)	Blood	Antibiotics (IV vancomycin and rifampicin for 6 weeks, gentamicin for the initial 2 weeks)	Discharged after clinical remission
Jacquier et al. [22]	F/50 L3/4 discectomy for sciatica 8 months previously Diabetes mellitus	Postoperative lumbar spondylodiscitis (fever and suprapubic pain for 1 day)	Biopsied bone	Antibiotics (IV ofloxacin and rifampicin for 2 weeks followed by oral therapy for 4 weeks)	Discharged after clinical remission
Hodiamont et al. [18]	M/40 Acute myeloid leukemia	Neutropenic fever with nosocomial pneumonia (neutropenic fever and right upper lobar pneumonia on day 19 after induction chemotherapy)	Blood, sputum and BAL fluid	Supportive treatment and antibiotics (IV ceftazidime, vancomycin, gentamicin and rifampicin)	Died on day 26 of admission due to multiorgan failure
	M/52 Acute myeloid leukemia	Neutropenic fever with nosocomial pneumonia (neutropenic fever and multilobar pneumonia on day 16 after induction chemotherapy)	Blood and BAL fluid	Supportive treatment and antibiotics (IV ceftazidime, vancomycin and rifampicin)	Died on day 27 of admission due to multiorgan failure
Nagler et al. [19]	M/68 Acute myeloid leukemia	Neutropenic fever with folliculitis and nosocomial pneumonia (neutropenic fever, scattered crusted papules in the groin and pneumonia on day 12–13 after induction chemotherapy)	Skin biopsy and BAL fluid	Antibiotics (IV cefepime and vancomycin)	Died on day 22 after induction chemotherapy
Blennow et al. [20]	F/43 Acute myeloid leukemia	Neutropenic fever with bacteremic pneumonia (neutropenic fever, right upper and middle lobar pneumonia 10 days after chemotherapy)	Blood and BAL fluid	Antibiotics (IV piperacillin/tazobactam, vancomycin, linezolid and co-trimoxazole)	Discharged after clinical remission
Present case	M/45 Left shoulder dislocation with tendon reconstruction	Implant-related septic arthritis and chronic osteomyelitis (recurrent left shoulder wound discharge for 5 years)	Debrided bone and wound tissue	Surgical debridement, removal of prosthesis, and antibiotics (oral doxycycline for 6 weeks)	Discharged after clinical remission

*F* Female, *M* male, *MVR* mitral valve replacement, *AVR* aortic valve replacement,*TOE* transoesophageal echocardiogram, *VP* ventriculoperitoneal, *TTE* transthoracic echocardiogram, *CSF* cerebrospinal fluid, *BAL* bronchoalveolar lavage, *IV* intravenous, *ICU* intensive care unit

In terms of antibiotic susceptibilities, *K. schroeteri* is frequently resistant to penicillin G (unlike micrococci) and oxacillin [9], a property which is considered to be specific for the genus *Kytococcus*. This is of clinical importance as these antibiotics are often used as first-line agents in cases of musculoskeletal infections caused by coagulase-negative staphylococci and micrococci. In such cases, empirical antibiotics consisting of ampicillin and cloxacillin are often used. The exact antibiotic susceptibility patterns are frequently omitted unless there is suboptimal clinical response to empirical treatment. As an illustration of this, the chronicity and the extent of disease involvement in our patient would probably have been less if further identification and antibiotic susceptibility tests had been performed on the initially isolated “*Micrococcus* spp.” to optimize the choice of antibiotics, rather than at the time when the patient suffered from progressive disease despite treatment with cloxacillin. Due to the scarcity of cases, there is no standardized guideline for the optimal choice of antibiotics in *K. schroeteri*-related infections. Because of the frequent occurrence of prosthesis-related infections in the reported case series, glycopeptides, namely, vancomycin and teicoplanin, in combination with aminoglycosides and rifampicin, were often required in the initial parenteral phase. Oral antibiotics, including fluoroquinolones and tetracyclines, with or without the addition of rifampicin, were used as oral maintenance depending on the individual strains’ susceptibilities, clinical severity, and the presence or absence of prostheses.

In summary, in our case report we describe two important novel findings of *K. schroeteri*: the existence of variable colonial morphologies and its capability to cause chronic implant-related joint and bone infection. Other clinical entities commonly associated with *K. schroeteri* include prosthetic valve endocarditis and fatal pneumonia in immunocompromised patients. Therefore, in patients who have prosthesis or are immunocompromised, further analysis to identify this potentially pathogenic organism using 16S rRNA gene sequencing should be performed after initial isolation of coagulase-negative staphylococci or micrococci in the laboratory. Treatment consisting of appropriate antibiotics and removal of infected prostheses is often necessary to prevent significant morbidities and mortalities.

## Nucleotide sequence accession number

The 16S rRNA gene sequences of the morphotypes have been lodged within the GenBank sequence database under Accession Numbers JF514888-JF514890.
